# The relation between school nurses’ professional role, jurisdictions and health promotion in Finnish compulsory schools: a qualitative study

**DOI:** 10.1177/17449871261469428

**Published:** 2026-09-15

**Authors:** Frida Gädda, Christel Sundqvist, Anna K Forsman

**Affiliations:** Doctoral Researcher in Health Sciences, The Faculty of Science and Engineering, Department of Natural and Health Sciences, Åbo Akademi University, Vasa, Finland; Professor, Faculty of Arts and Social Sciences, Department of Educational Studies, Karlstad University, Karlstad, Sweden; Senior Lecturer in Special Pedagogy, The Faculty of Human and Social Sciences, Department of Pedagogy, Åbo Akademi University, Vasa, Finland; Associate Professor and Senior Lecturer in Health Sciences, The Faculty of Science and Engineering, Department of Natural and Health Sciences, Åbo Akademi University, Vasa, Finland

**Keywords:** compulsory school, health promotion, jurisdictions, school healthcare, school nurse, qualitative research

## Abstract

**Background:**

School nurses are a vital part of school healthcare; however, research on the experiences of the profession in relation to health promotion is limited. A deeper understanding of school nurses’ professional remits could uncover new approaches to school-based health promotion – with implications for student healthcare services.

**Aim:**

This study explored school nurses’ experiences regarding their professional role and responsibilities in relation to health promotion in Finnish compulsory school settings.

**Methods:**

Adopting a qualitative design, 13 school nurses from Finland were interviewed. Data analysis followed qualitative content analysis, progressing from manifest to latent content, explored through Abbott’s theory of system of professions.

**Results:**

The main categories: *The practical application of laws and regulations shaping preventative actions*; *Societal changes and resources demanding continuous adaptation*; *School nurses providing accessible, holistic support and health promotion*; and *Individual autonomy, interests and visions influencing the work*, pinpoint the school nurses’ experiences regarding their jurisdictions related to health promotion, impacting the school nursing services.

**Conclusion:**

School nurses’ jurisdictions in health promotion are shaped across legal, workplace and public arenas. An additional individual-level arena was found, where ethics guide practice. Structural constraints require school nurses to adapt as demands extend beyond traditional boundaries to meet students’ needs.

## Introduction

School healthcare services are defined as services targeting students in primary or secondary education, provided by a health professional inside or outside school (World Health Organisation (WHO) and United Nations Educational, Scientific and Cultural Organisation (UNESCO), 2021). In Finland, these services aim to support students and their families’ health and well-being, while simultaneously promoting students’ growth and development (*Terveyden ja hyvinvoinnin laitos*/Finnish Institute for Health and Welfare (THL), 2025a). Thus, school healthcare services refer to services provided by various occupational groups within healthcare, for example school nurses (hereafter SNs), physicians, counsellors and psychologists. This study focuses specifically on the SNs’ role within Finnish school healthcare services.

Health promotion is defined by WHO (1986; 1998) as enabling individuals to take control over and improve their health and since schools reach large numbers of students and families, they are considered ideal arenas for health-promoting initiatives (WHO, 2017). Consistent with the Ottawa Charter (WHO, 1986), SNs are uniquely positioned for these initiatives, offering accessible health services in schools. Finland and other Nordic countries have regulated the SNs work through legislation (Lag om elev- och studerandevård, 2013; Forskrift Om Helsestasjons-Og Skolehelsetjenesten, 2018; Hälso- och sjukvårdslag, 2010; Skollag, 2010; Sundhedsloven, 2005). In a Finnish context, the SN practice’s primary focus should be on the health and well-being of students and their families, aiming to promote and sustain optimal health and healthy school environments, thereby supporting the students’ educational development fostering them to healthy citizens (THL, 2025a). The work performed by a public health nurse is oriented towards promotion and prevention, aiming to create and support the conditions for health while preventing diseases. The services are provided for students in grade 1–9 in compulsory schools and their families (Finnish Social- and Health Department and STAKES, 2002; THL, 2025a; Hälso- och sjukvårdslag, 2010). The recommended staff resourcing for Finnish SNs is set in relation to the number of students, with a ratio of 460 students per full-time nurse, which means SNs often work in multiple schools (THL, 2025b).

Professional work and development are seen as a dynamic and complex relationship shaped by continuously changing landscape of tasks, professions and interprofessional interactions (Abbott, 1988). These changes are influenced by both internal dynamics and broader social and cultural forces. However, there is no universally agreed definition of SNs’ responsibilities, and their scope of practice varies across countries due to differences in educational and healthcare systems (Baltag et al., 2015). Despite these variations, the occupations working close to SNs all recognise the importance of SNs within schools (Bergren and Maughan, 2017). Previous research highlights ambiguity surrounding SNs’ societal role and role in the school setting (Maughan and Adams, 2011; Reutersward and Hylander, 2017; Winland and Shannon, 2004) and challenges in defining their professional remit have been identified globally (Bergren and Maughan, 2017; Houlahan, 2018; Reutersward and Hylander, 2017). Deeper understanding of SNs’ experiences of their work and health promotion offers potential to address the ambiguity regarding the SNs’ professional role and uncover innovative health promotion strategies applicable for SNs when supporting students in creating healthy lifestyles. Research shows that school nursing services help reduce healthcare costs, improve student attendance and enhance teacher and staff productivity (Wang et al., 2014) but further studies on SN services are required (Pawils, et al., 2023). More than a decade ago, Baltag et al. (2015) called for countries to analyse the school healthcare services provided and align its scope within the evidence base, considering national health matters. There is growing concern over the deteriorating physical and mental health of today’s children and adolescents (The Organisation for Economic Co-operation and Development (OECD) and European Commission, 2024); and knowledge about how this affects the work of SNs in Europe and Finland is limited. In addition, empirical research exploring Finnish SNs’ role as health promoters, including their own experiences, remains limited, thereby providing the rationale for this study.

Abbott’s theory on the system of professions (Abbott, 1988) is used as theoretical framework. Abbott (1988) introduced the concept of *jurisdiction* to explain how professions are linked to their work, arguing that professional development depends on how this connection is established, maintained and modified across various formal and informal levels. Jurisdictions are influenced by public, legal and workplace arenas, as well as by broader social and cultural structures. Historically, jurisdictional relationships between professions have evolved through jurisdictional claims (Abbott, 1988). Through the process of jurisdictional claims, a profession gains jurisdiction over new areas of work. A change in one profession’s jurisdiction will affect jurisdictions of surrounding professions, as they are all interconnected within a system (Abbott, 1988). There are a few studies examining nursing through the lens of Abbott’s theory (Johannessen, 2018; Manley, 1995; Thompson, 2008) mainly focusing on the tension between nurses and physicians. To our knowledge, the theory has not previously been used to examine the SN profession. This theory thus provides a lens through which to explore how SNs’ jurisdictions are shaped within the school context and through interactions with other professionals and students. Exploring SNs’ practical experiences of their work can therefore provide valuable insights into professional legitimacy and development.

## Methodology

### Aim and research question

The aim of this study is to gain a deeper understanding of SNs’ experiences about their professional jurisdictions, role and responsibilities in relation to health promotion in Finnish compulsory schools. The research question is: How do SNs experience the relation between their professional role and school health promotion?

### Design and setting

As the aim was to gain a deeper understanding of SNs’ experiences, this study adopts a qualitative design, using semi-structured interviews for data collection.

### Participants and recruitment

Participants were recruited through school nursing department managers in the selected well-being service county. Information was distributed to the managers by the first author (FG) who then met with staff providing information about the study. Interested SNs contacted the first author directly via email and provided written informed consent prior to participating. Inclusion criteria were Swedish-speaking SNs with at least 1 year of experience as an SN in the selected well-being service county. The health- and social care reform taking place in Finland in 2023 created challenges in participant recruitment, extending the process.

### Data collection

Data collection took place in 2022–2024 through individual semi-structured interviews with 13 Finnish SNs working in compulsory schools targeting students at ages 6–16 years. The interviews took place online (*n* = 11) and face to face (*n* = 2) and were recorded and transcribed verbatim. The interviews lasted 45–90 minutes, and transcripts counted for a total of 140,330 words on 334 pages. All interviews were conducted and transcribed by the first author (FG). The interview guide (see Supplementary material) consisted of different themes, focusing on the SNs views and experiences of their work, the connection between their work and health promotion, as well as interprofessional collaboration.

### Data analysis and trustworthiness

Qualitative content analysis by Granheim and Lundman (2004) and Granheim et al. (2017) was used for data analysis, with inspiration from Lindgren et al. (2020) in interpretation of the latent content. The analysis was conducted in two phases. Firstly, an inductive, data-driven approach was used, examining the manifest content of the transcripts. The first author (FG) kept notes during the preliminary reading, created meaning units and coded the data. The initial understanding of the data was discussed with the co-authors (CS, AKF) before continuing the analysis process. In the second phase, the research team conducted an iterative process involving organising the codes and creating subcategories and categories until consensus was reached. The system of professions (Abbott, 1988) was used as theoretical framework when interpretating the manifest subcategories into latent main categories, especially focusing on the concept of jurisdiction and how this can be described in relation to the SNs’ profession. In line with Lincoln and Guba’s (1985) credibility criteria for trustworthiness, the categories were finally discussed and examined in relation to both the theoretical framework and the transcripts. Reflexive notes were kept from the discussions to strengthen dependability. The authors strived to include participants of varying ages and experiences ensuring transferability and to further strengthen conformability, quotes were used to highlight SNs’ experiences. A sequence from the analysis is visualised in Table 1.

**Table 1.** Sequence from analysis process.

Meaning unit	Condensation	Code	Subcategory	Main category
*And one of the biggest tasks we have is that a large part of our work is determined by law. It is something we must do; we cannot simply ignore it. These are our health checks, and there are both recommendations and laws governing how they should be carried out, and we must comply with these as far as we can. Then there is the completely different issue of a lack of resources and our inability to comply with all the points in the law and the recommendations. And then we need to make new decisions about how this school should be run during this term or during this school year. (R9)*	*A large part of our work us is required by law. . . These are our health checks, and there are both recommendations and laws governing how they should be carried out, and we must comply with these as far as we can. Then there is the completely different issue of. . . our inability to comply with all the points in the law and the recommendations.*	The biggest task is statutory health checks governed by law and recommen-dations	Statutory health examinations	The practical application of laws and regulations shaping preventative actions (formal jurisdiction)

### Ethical considerations

Ethical approval for the study was granted on 29 November 2021 by the Board for Research Ethics at Åbo Akademi University. Research permits were also granted [378/13.01/2023] from the well-being service county. The participants were informed about the study’s purpose, methodology, voluntary approach and data management prior to participating and informed consent was obtained from all participants prior to the interviews. The collected data were pseudonymised, and the SNs were assigned identifiers to ensure confidentiality. The COnsolidated criteria for REporting Qualitative research (COREQ)-protocol (Tong et al., 2007) was followed and is included in Supplementary material 2.

## Results

All participants (*n* = 13) were registered nurses and public health nurses, with work experience ranging from <5 years to >20 years. They were working for a well-being service county in Finland and located in compulsory school settings, responsible for 1 up to 4 schools, encountering in between 150–450 students each. All participants were female; however, this reflects the national context, as 99.4% of public health nurses in Finland were female in 2023 (THL, n.d.). Participants’ characteristics are found in Table 2. The collected data describe the SNs’ experiences of the breadth and depth of school nursing and how this can be connected to health-promotion initiatives. Created subcategories and the main categories are shown in detail in Table 3 below.

**Table 2.** Characteristics of participants.

Respondent	Age group^a^	Work experience as SN (years)	School (*n*)
R1	B	<5	2
R2	B	<5	2
R3	B	<5	2
R4	C	5–10	4
R5	B	>10	2
R6	C	>10	1
R7	C	>10	2
R8	A	<5	3
R9	B	5–10	—
R10	B	5–10	—
R11	B	>10	2
R12	B	>10	2
R13	A	<5	2

A dash (—) indicates missing data.

SN: school nurse.

a Age groups: A = <30, B = 30–50, C = >50.

**Table 3.** Manifest sub-categories forming latent main categories.

Manifest subcategories	Latent main categories
Focus on physical health separates SNs from other school healthcare personnel	The practical application of laws and regulations shaping preventative actions (formal jurisdiction)
Preventive work for all students, for example health examinations and vaccinations	
First aid, emergency care, health and medical care	
Environmental inspections of the school	
Lessons, classroom activities and public events	
Role changing over time due to societal changes and needs	Societal changes and resources demanding continuous adaptation (formal jurisdiction)
Great need for addressing mental challenges, overlapping counsellors/school psychologists work	
Resources and time constraints creating a difficulty in prioritisation	
Broad area of work, seeing the whole human being	SNs providing accessible, holistic support and health promotion (informal jurisdiction)
Visibility, availability and presence in school necessary	
Low-threshold work	
Health promotion	
Individual and lonely work, demanding flexibility and planning whilst creating a fear of missing something	Individual autonomy, interests and visions influencing the work (informal jurisdiction)
Task diversity needed for the job to be interesting	
Creating trust and safety	
Supporting students and families, being on the students’ side	

SN: school nurse.

### The practical application of laws and regulations shaping preventative actions (formal jurisdiction)

*Category one* describes the dimensions of school nursing as regulated by legislation (Hälso- och sjukvårdslag, 2010) and national recommendations (THL, 2025a) in Finland. According to the SNs, what distinguishes their role from other school healthcare personnel is their annual contact with all students and their stronger emphasis on physical health:

*I’m more responsible for that specific, physical bit where you do all the measurements and follow up on everything. No one else does that. Of course, you’re also there, like all the other adults, as psychological support, but it’s the same for basically everyone who works with the children, to make sure that they are psychologically and psychosocially well. So, the physical part is perhaps what is most distinctive, no one else works with that*. (R8)

Preventative work, such as health examinations, emergency care, vaccinations, measurements and discussions about lifestyle habits are described as a central part of SNs’ responsibilities. Additional duties include being part of classroom or school activities related to health education and regularly assessing the school environment. Participants also emphasise a lack of sufficient time for these activities:

*The main thing is that we work preventively, through these statutory health examinations and follow-ups that are part of the statutory preventive work. Follow-ups on both physical, mental health and well-being [. . .] Then of course there is collaboration with the school, you can go into the class and inform about different things and maybe participate in health education lessons. Unfortunately, in practice there isn’t enough time for that, even though I think it should be a part of it too*. (R5)

The regulations allow for a degree of interpretation when implemented into practice. For example, participants addressed treating illnesses in schools with mixed reactions. On one hand, they argued that their responsibility should be limited to providing emergency care in cases of accidental injury. On the other hand, they described caring for all the minor health concerns as a potential enabler of addressing more complex issues later. Participants found it challenging to refrain from managing illness in school healthcare, as symptoms may indicate more than a temporary condition:

*Now we should remove treating illnesses altogether [. . .] It’s a shame — through these small encounters, you get to know a child because they are brave enough to come, and when you get to the bottom of it, it turns out to be something completely different. So, it’s these small concerns that you need time for, as they help you establish contact*. (R4)

### Societal changes and resources demanding continuous adaptation (formal jurisdiction)

*Category two* addresses how the work is impacted by traditional values and perceptions of the SN profession, even though the role has evolved and requires continuous adaptation to be able to respond effectively to societal and cultural changes: *If you think about school health services in the past, it could be one in ten that had a problem, now it’s one in ten that have no problem, that’s has changed and that’s what is so time consuming* (R11).

Participants highlight that their work has shifted towards caring for students’ mental and social issues, such as giving psychosocial support. In several cases, this overlaps with the school counsellors’ and psychologists’ responsibilities, particularly when resources are limited or the student are not yet ready to visit other mental health professionals inside or outside the school environment:

*There could be situations, especially between me and the counsellor, like we were a bit into each other’s clients, so to speak. But we talked about it [. . .]. I was there more often than the counsellor, so it was more natural for the child to come to me and talk or sit or whatever [. . .] I think it’s very important that you as a SN have an open dialogue with the counsellor*. (R9)

Although more services are provided within school healthcare, additional resources for the extended responsibilities are not available. The SNs emphasise that Attention Deficit/Hyperactivity Disorder (ADHD) follow-ups occupy an increasing proportion of their time, as such cases require a family centred, multi-collaborative approach. They experienced being continuously assigned new tasks in addition to their statutory responsibilities. This is putting a considerable strain on their work, as resources are allocated according to the regulations on students’ annual statutory health examinations, rarely considering other responsibilities. Participants reported having to prioritise and leave out some of the statutory health examinations due to shortage of time and resources. The SNs’ experience that their work is given lower priority than that of other school and healthcare professionals, often requiring them to reschedule visits or assist others:

*Yes, it takes a lot of time, and I have often emphasized that to the management when I have made my presentations about more resources. There is so much of this work that they do not know, usually they know routine health examinations, figures, visitor statistics and such, but they do not see… we are in a way the spider in the web all the time in different areas, which they do not know*. (R10)

With new demands come the need for new methods; however, SNs report insufficient time to learn and implement these in their daily practice. The SNs expressed a desire for more time to adopt new methods, maintain parental contacts and offer open-door clinics, enabling students to make spontaneous visits when necessary.

### SNs’ work providing accessible and holistic support (informal jurisdiction)

*Category three* explores SNs’ experiences of their health-promoting role in schools. The SNs described being primarily associated with addressing students’ physical health, but all emphasised the importance of adopting a holistic approach in caring for students, considering the physical, mental, spiritual and social factors impacting the students’ life and health, and responding to these accordingly: *You must see the whole person [. . .] even sad children, angry children and happy children, you should learn to read them a little on how they feel. You must listen and tune in. That’s probably our tools really…that you take time if necessary* (R4).

Participants reported a need to be prepared for almost anything, as they usually are the first point of contact for students, guardians and school staff needing support. Therefore, they feel a need for broad and extensive knowledge to support their professional role. Participants emphasised their efforts to incorporate health promotion into every encounter, regardless of the reason for which the student seeks SN services; ‘*I don’t think you can study to be a SN, you are one from the start [. . .] either you have that ‘thing’ where you get health promotion into everything, or you don’t*’ (R6). SNs express a desire to be more involved in general health promotion not only within the school environment but also in the individual encounters.

Participants noted that their role differs from other school staff, as they do not hold disciplinary responsibilities. This distinction allows students to discuss difficult matters openly with the SN without fear of reprimand, thereby creating a safe environment for dialogue. The SNs described the importance of being visible in the school setting and providing a low-threshold service that students and guardians can easily access for support and guidance: ‘*Both in terms of teachers, guardians and students, I would say that the threshold is always lowest to the SN*’ (R6). Low-threshold services aim to provide easily accessed help and support, often free of charge and closely situated. SN services are seen as important low-threshold service for students and their families. However, the low-threshold service is dependent on SNs’ presence in the school environment, preferably several days a week, as well as availability through other channels, such as online chat or telephone. Participants responsible for multiple schools described this as challenging, whereas SNs present full time in a single school reported that students were referred to them more frequently.

### Individual autonomy, interests and visions influencing the work (informal jurisdiction)

*Category four* highlights individual factors associated with SNs’ health promotion and healthcare work. All participants described their work as independent yet lonely, perceived both positively and negatively. Although SNs have autonomy over their daily work and schedule, this independence also brings isolation and responsibility for planning and completing statutory tasks by the end of the school year, as they are the only professionals performing these tasks. Participants noted that their work often goes unnoticed, as they work behind the scenes to support students and families: *I guess that’s the thing about SNs and school health services, we don’t have to be at the front, we’re quite used to getting on with it. If we get praise and compliments, we’re delighted, but when we succeed with a student, that’s our reward* (R4).

SNs desire more variation in their work. Only performing routine health examinations gets repetitive; therefore, they enjoy the possibility to impact their schedule to find tasks that motivates them. Given the highly autonomous nature of their work, SNs are offered opportunities to engage in tasks they perceive meaningful and important. This also applies to general health-promoting initiatives in schools, since interest to engage in these activities was expressed, whilst some participants expressed that they would need more pedagogical education and experience to feel secure in delivering general health-promoting initiatives.

SNs strive to maintain a family- and student-centred approach, safeguarding children within the school context, describing flexibility in providing necessary services and sufficient time for each student as essential to their health-promoting role. Taking time and being adaptable were key factors in building a relationship with the student and creating trust and safety. This approach creates a strong sense of responsibility to attend to all students, as illustrated in the following quote: ‘*SNs are concerned that they might overlook something, but they need to be supported in understanding that it is ultimately the guardians who have the primary responsibility*’ (R10). Supportive colleagues and collaborative practices were highlighted as crucial in managing these feelings and preventing professional isolation.

## Discussion

This study shows that SNs’ work is significantly influenced by societal changes and trends affecting students’ health, creating tensions with both external and internal frameworks, and with other professionals. This means that the traditional SN role requires revision to reflect contemporary demands, as SNs are advancing and expanding their jurisdictions. Another finding is that regulatory frameworks determine the extent to which SNs can engage in general health promotion within the school. Although these frameworks aim to ensure equitable services for all students, they simultaneously restrict opportunities for broader health-promoting initiatives. Consequently, SNs must adapt and reprioritise activities in response to students’ needs, often requiring them to operate beyond the system’s structural boundaries. The results illuminate the complex and broad interprofessional areas that SNs work in, with different social and cultural factors affecting their jurisdictions in the formal arenas: *the legal system* and *public opinion*, as well as in the informal arena of *the workplace* (Abbott, 1988). Furthermore, the study reveals potential impact on jurisdictions from an additional informal arena, *the individual level*, containing the ethical and moral decisions internally affecting the SNs’ work and commitments especially to health promotion.

School nursing has historically had a broad responsibility within public health, with a primary focus on physical health and close collaboration with school physicians. To this day, SNs remain dependent on collaborative partnerships with physicians to deliver required services. This is not unique to school nursing, as nursing has historically been positioned as subordinate to medicine (Abbott, 1988). However, this study highlights other significant jurisdictional relationships between SNs and school counsellors and psychologists. The participants reported some overlap with the school counsellors’ responsibilities, particularly in providing mental and psychosocial support, which is becoming an increasingly prominent aspect of the SNs’ role, and which SNs do not necessarily feel equipped to address (Bekaert et al., 2025; Putkuri et al, 2021). This finding points to tensions between the professions jurisdictions (Abbott, 1988) and the overlap necessitates effective communication and clear division of labour within school healthcare services (Abbott, 1988). Further research could explore these tensions due to the potential impact on health promotion. One explanation for this, is that SNs are the only professionals in compulsory schools who meet every student annually, thereby continuously strengthening the nurse–student relationship. The results also indicate responsibilities previously managed within Finnish specialist care, such as ADHD-assessments and medications, are increasingly being transferred to school health services. This shift occurs without provision of adequate resources or training, thereby redirecting SN focus from health promotion and prevention towards more individualised healthcare. Viewed through the lens of Abbott’s theory (Abbott, 1988), these developments illustrate how changes in professional jurisdictions, driven by multiple factors, influences the entire professional system.

SNs hold a specialist role in public health (Cook et al., 2023; Sammut et al, 2022), working at a broad level and collaborating with students and their families, health and social care providers, as well as educational partners. To contribute effectively to health promotion, SNs require adequate education and resources (Jakobsson and Moberg, 2025). Results from this study and previous Nordic research (Jakobsson and Moberg, 2025; Kletthagen and Moen, 2017; Morberg, 2025), agree that SNs play a vital role in health promotion within schools. They strive to promote health holistically, recognising the strong interconnection between health and learning (WHO, 2014). The vision of SNs’ health-promoting role appears to conflict with the caseload approach (Morse et al., 2022) to resource allocation. Furthermore, SNs are often responsible for several schools, reducing their physical presence at each site. This situation can be interpreted as a conflict of interests between SNs’ perceptions of their desired professional jurisdiction and system-level constraints imposed by legal frameworks and workplace structures (Abbott, 1988), particularly regarding accessible health promotion.

In line with the Ottawa Charter (WHO, 1986), SNs have a unique position in health promotion by providing free services to all students, regardless of socioeconomic status, which also support their educational achievements. Previous research indicates that capacity to deliver health promotion is influenced by the individual SN (Jakobsson and Moberg, 2025; Reutersward and Lagerstrom, 2010). However, all SNs in this study, expressed a desire to integrate health promotion into every student encounter. To maximise SNs’ health-promoting role, they need sufficient time and resources to create a safe space accessible to all students and guardians (Kletthagen and Moen, 2017). Participants emphasised that facilitating access to low-threshold services, such as open-door clinics, enables SNs to become acquainted with students through brief interactions, that prove valuable later when more complex issues are addressed. This is supported by earlier research (Kletthagen and Moen, 2017) regarding SNs’ mental health-promoting role. SNs in this study reported that time for such services is limited, as health examinations, mental health problems and administrative duties occupy their time.

Although both this study and previous research (Flodin et al., 2025) indicate that SNs perceive some autonomy in their work, they also report stress due to their limited opportunities to engage in general health promotion in schools, constrained by the volume of mandatory health examinations. Previous research (Michaud et al., 2021) has argued that school health services would benefit from extending the remit beyond screenings and vaccinations. Nevertheless, SNs emphasised the importance of meeting each student annually to assess their well-being for early identification of potential health concerns. This creates a paradox, posing moral and ethical dilemmas potentially causing work-related stress and isolation. The highly individualised nature of school nursing, combined with significant time constraints often compels SNs to prioritise statutory, individual-level duties at the expense of general health-promoting initiatives. Such discretionary decision-making may influence the professional jurisdictions. This autonomy may lead to variations and inequities in health-promoting services delivered to students across schools nationwide. Abbott (1988) focused on the systemic level of how jurisdictions and professions are constituted, rather than the individual, ethical and moral dimensions shaping their development. However, he notes that professions operate within their own ethical codes, a principle that also applies to Finnish public health nurses (The Finnish Association of Public Health Nurses, n.d.). Therefore, this finding could be considered a potential extension of the theoretical framework, adding an additional informal dimension to the theory on how jurisdictions are affected by the individuals’ prioritisation and how the professionals’ own moral and ethical compass guide and protect their work.

SN practice is closely aligned with the evolving health challenges of society (Morberg, 2025). With the rapid societal changes as well as the growing prevalence of mental health problems among young people (OECD and European Commission, 2024), legislative and policy frameworks regarding SNs’ work struggle to keep pace. Consequently, SNs in collaboration with their managers need to navigate between sharing and claiming new tasks while simultaneously acquiring enough competence and resources to perform these tasks. Finnish SNs express a strong commitment to providing high-quality care but feel constrained by both formal and informal jurisdictional boundaries, a finding supported by research from other Nordic countries (Flodin et al., 2025; Jakobsson et al., 2026; Jakobsson and Moberg, 2025). As health promotion implies enabling people to take control of their own health (WHO, 1986), SNs are an important resource, but their role is fragmented, as they are expected not only to work autonomously with individual students, preventing ill health and promoting healthy lifestyles, but also to advocate for health within the wider school setting. However, the results from this study, supported by earlier research (Jakobsson and Moberg, 2025), show that broader health-promoting activities and larger coordinated actions are somewhat limited by jurisdictional constraints and lack of resources. This requires the SNs to be flexible and creative in delivering their services while balancing limited resources with diverse and evolving demands.

### Strengths and limitations

Data were collected during Finland’s health and social services reform in 2022–2023, which may have influenced the findings. The SNs’ employer changed from municipality to well-being services county, changing the organisational structure while the work remained legally regulated. Although not directly addressed in the interviews, the reform was mentioned by participants; however, as it was not the study’s focus, no conclusions can be drawn about its impact on the SNs’ work.

FG, who conducted the interviews and coded the data, is a qualified public health nurse with prior experience as an SN, which can be regarded as both a strength and a limitation. It nonetheless facilitated deeper engagement during the interviews and was transparently discussed within the research team and with participants. The study includes 13 participants, working within one well-being services county in Finland. The sampling strategy entails a risk of self-selection bias but was deemed appropriate for exploring SNs’ professional experiences through voluntary participation. The sample included SNs of varying ages and experience across the well-being service county and support diverse perspectives in line with Lincoln and Guba’s (1985) concept of transferability. As school nursing practice varies across countries, the findings may not be directly transferable; however, given the similarities within Nordic contexts, the results may be applicable in these settings. Nevertheless, the study identifies factors related to the profession and health promotion that can be adapted to support SNs internationally.

## Implications

The findings indicate that revising the Finnish SN role description is necessary to reflect emerging clinical demands, while simultaneously balancing traditional responsibilities. As SNs expand their jurisdictions, national policy development is needed in both school nursing and school-based health promotion to clarify and strengthen SNs’ role and legitimacy. Furthermore, this study underscores the importance of recognising SNs as key players in school health promotion, emphasising that their ability to carry out these tasks depends on adequate resourcing. Role refinement also requires corresponding updates to nursing curriculum to ensure alignment between school nursing education and clinical practice. Further research could explore tensions between SNs and school counsellors and psychologists that may impact school health promotion and examine user perspectives on school health services, particularly students’ needs.

## Conclusion

The findings suggest that SNs’ jurisdictions in health promotion re-affected on all arenas mentioned in Abbott’s theory of system of professions (Abbott, 1988), *the legal, public opinion and the workplace*. Societal changes and health trends significantly shape the work, creating tensions with regulatory frameworks and other professional groups, particularly regarding SNs’ capacity to engage in general health promotion. The results highlight *the individual level* as an additional informal arena of jurisdiction, where ethical and moral decisions of the individual SN may influence their workload and health-promoting activities. Although systemic frameworks aim to ensure equality, they shape SNs’ capacity to engage in general health promotion. This requires that SNs frequently adapt and operate beyond structural constraints to meet students’ needs.

**Key points for policy, practice and research**

- Societal changes and health trends influence SNs’ work, creating tensions with external and internal frameworks, ethical guidelines and other professionals, thereby highlighting the need for role development.
- Regulatory frameworks guide SNs towards providing equivalent services but simultaneously shape their capacity to engage in broader health-promotion initiatives, posing challenges in balancing traditional expectations of their role with new evolving demands.
- An updated SN role description is essential to address emerging clinical requirements, understand and affirm the SNs’ central role in school health promotion and ensure they have adequate resources to perform their work as a low-threshold service for students.

## Supplemental Material

sj-docx-1-jrn-10.1177_17449871261469428 – Supplemental material for The relation between school nurses’ professional role, jurisdictions and health promotion in Finnish compulsory schools: a qualitative study Supplemental material, sj-docx-1-jrn-10.1177_17449871261469428 for The relation between school nurses’ professional role, jurisdictions and health promotion in Finnish compulsory schools: a qualitative study by Frida Gädda, Christel Sundqvist and Anna K Forsman in Journal of Research in Nursing

sj-docx-2-jrn-10.1177_17449871261469428 – Supplemental material for The relation between school nurses’ professional role, jurisdictions and health promotion in Finnish compulsory schools: a qualitative study Supplemental material, sj-docx-2-jrn-10.1177_17449871261469428 for The relation between school nurses’ professional role, jurisdictions and health promotion in Finnish compulsory schools: a qualitative study by Frida Gädda, Christel Sundqvist and Anna K Forsman in Journal of Research in Nursing
