# Supplementary material for: The relation between school nurses’ professional role, jurisdictions and health promotion in Finnish compulsory schools: a qualitative study
Source: J Res Nurs. 2026 Sep 15:17449871261469428. Online ahead of print. doi: 10.1177/17449871261469428 (PMC13582656; doi:10.1177/17449871261469428)
Supplement: Supplementary file 1 [file sj-docx-1-jrn-10.1177_17449871261469428.docx]

# Appendix 1. Interview guide

**Section 1: Background Information**

1. Could you please briefly describe your current professional role
   1.1 How many schools do you currently work in, and approximately how many students do you serve? What age groups do these students represent?
   1.2 Could you outline your professional experience, including the duration of your work as a school nurse or in related fields?
   1.3 What are your primary work tasks? Do you have any additional

responsibilities or areas of practice (e.g., student health services, maternity care, child health services)?

2. If you are comfortable sharing, please provide the following background information: age, educational background, year of qualification as a public health nurse, salary, and gender.

# Section 2: General Perspectives on School Nursing and Health promotion

1. How would you describe the main responsibilities and areas of work associated with school nursing?
2. How would you describe health promotion?
   3.1 How do you as a school nurse engage in health-promoting activities?
   3.2 In what ways does your health promotion work differ from that of other school staff?

# Section 3: General perspectives on collaboration

1. With whom do you collaborate within and outside the school setting?
   1.1 Are there any stakeholders with whom you would like to strengthen collaboration?
   1.2 What types of interactions do you have with school staff, teachers, and students in your daily work?
2. What other student welfare services are available at your school, and how would you describe your collaboration with them?
   2.1 How do you perceive the division of responsibilities within student welfare services?
3. How do you perceive the role of school nursing within student welfare services and the broader school environment?

# Section 4: Good Practices

1. What aspects of school nursing work well? Especially in relation to health promotion?
2. What characterizes effective interprofessional collaboration in your experience? Please provide examples.
   2.1 What factors facilitate effective collaboration?
3. Are there formal policies, directives, or guidelines that support collaboration between school staff and school health services, school nurses in your organization?
   3.1 If formal collaboration guidelines are lacking, do you believe they are needed? Please explain your reasoning.

# Section 5: Challenges

1. What challenges do you encounter in your work as a school nurse?
2. What challenges are associated specifically with health promotion?
3. Are there factors that hinder or negatively affect collaboration? Please provide examples.
4. What measures could help prevent or reduce barriers in health promotion or collaboration?

# Section 6: Development Priorities and areas for improvement

1. What areas for improvement exist within school health services, particularly in health promotion?
   1.1 How could health-promoting work be strengthened in schools today?
2. What are the current needs regarding collaboration in your work?
3. What aspects of collaboration could be further improved?
   3.1 What could strengthen collaboration between school staff and school health services/school nurses?
   3.2 What could improve collaboration between students/parents and school health services/school nurses? What changes would be necessary to facilitate smoother collaboration?
4. What future challenges do you anticipate in school nursing practice?

**Closing question:** is there anything further you would like to add that has not been covered but that you consider important?
