# Supplementary material for: The relation between school nurses’ professional role, jurisdictions and health promotion in Finnish compulsory schools: a qualitative study
Source: J Res Nurs. 2026 Sep 15:17449871261469428. Online ahead of print. doi: 10.1177/17449871261469428 (PMC13582656; doi:10.1177/17449871261469428)
Supplement: Supplementary file 2 [file sj-docx-2-jrn-10.1177_17449871261469428.docx]

# Appendix 2.

COREQ 32-item checklist for interviews and focus groups (Tong, Sainsbury & Craig, 2007).

| **Research team and reflexivity** |  |  |
| --- | --- | --- |
| 1. | Interviewer/facilitator | FG conducted all the interviews |
| 2. | Credentials | FG = PhD candidate AF = DrPH  CS= PhD |
| 3. | Occupation | FG = Doctoral Candidate, Public Health Nurse, Registered Nurse AF= Associate Professor  CS = Professor, Senior University lecturer |
| 4. | Gender | All of the researchers are female. |
| 5. | Experience and training | FG = Has experience from planning and conducting several interviews as well as practical experience from the clinical field as public health nurse  AF= Has experience from planning and conducting multiple interviews and led national and international research projects on health promotion contexts and approaches.  CS= Has experience from planning and conducting multiple interviews and national and international research projects on special education and pedagogy context |
| **Relationship with participants** |  |  |
| 6. | Relationship established | FG was employed by the welfare county in question during this study; however she was not in an active working relationship due to study leave for this doctoral project. The data collection was for this reason not conducted in the same city as FG had been  practicing. |
| 7. | Participant knowledge of the interviewer | The informants knew which organizations the researchers represented and their research interests in school nursing. |
| 8. | Interviewer characteristics | In the manuscript strengths and limitations section, the researcher’s (FG) preunderstandings as public health |

|  |  | nurse are highlighted as a potential bias. |
| --- | --- | --- |
| **Theoretical framework** |  |  |
| 9. | Methodological orientation and theory | Qualitative content analysis with a inductive approach |
| **Participant selection** |  |  |
| 10. | Sampling | Purposeful sampling, self-selection |
| 11. | Method of approach | Study information was circulated through emails to the school nurses’ heads of department and directly to potential participants through staff email lists. FG was invited to speak to potential  participants at a staff meeting. |
| 12. | Sample size | 13 |
| 13. | Non-participation | About 35 school nurses were contacted. |
| 14. | Setting of data collection | The interviews were conducted both online (n=11) and face to face (n=2). Face to face interviews took place at the participants wokplace. |
| 15. | Presence of non-participants | - |
| 16. | Description of sample | The participants represented school nurses working in compulsory schools in a welfare county. All study participants were women and their work experience varied between under five to over 20 years. Characteristics of participants are further described  in table 1. |
| **Data collection** |  |  |
| 17. | Interview guide | A semi-structured interview guide consisting of broad themes and related questions guided the interviews. The interview guide was pilot tested on two school  nurses before the interviews took place. |
| 18. | Repeat interviews | - |
| 19. | Audio/visual recording | The interviews were audio recorded. |
| 20. | Field notes | The interviewer made notes during the interviews. |
| 21. | Duration | The interviews lasted between 45-90 minutes. |
| 22. | Data saturation | Data saturation was discussed among the researchers after the 13th interview and the researchers  decided to not recruit more informants than already appointed. |
| 23. | Transcripts returned | - |
| **Data analysis** |  |  |

| 24. | Number of data coders | FG conducted the initial coding of the data and all authors  contributed to generating the themes. |
| --- | --- | --- |
| 25. | Description of the coding tree | Table 2 displays the analysis process from meaning unit to codes, subcategory and category. Table 3 shows the manifest subcategories and how these  formed the latent main categories |
| 26. | Derivation of themes | Example of the data analysis process are given in Table 2. |
| 27. | Software | Microsoft Word |
| 28. | Participant checking | - |
| **Reporting** |  |  |
| 29. | Quotations presented | The reporting of the findings includes quotations from the raw data (translated from Swedish to English by the authors) and each quotation can be linked to a  participant number |
| 30. | Data and findings consistent | The authors have set out to keep the data closely to the (manifest) in the first step of analysis and then deepen the interpretation of the latent content with help from the lens of Abbotts (1988) theory of the systems of professions. The process of looking into the latent content was continuously discussed within the research  team. |
| 31. | Clarity of major themes | The major themes are described in text as well as in Table 3. |
| 32. | Clarity of minor themes | The minor themes are described in the text as well as in Table 3. |
